# A functional model and simulation of spinal motor pools and intrafascicular recordings of motoneuron activity in peripheral nerve

**DOI:** 10.3389/fnins.2014.00371

**Published:** 2014-11-14

**Authors:** Mohamed N. Abdelghani, James J. Abbas, Kenneth W. Horch, Ranu Jung

**Affiliations:** ^1^Adaptive Neural Systems Lab, Department of Biomedical Engineering, Florida International UniversityMiami, FL, USA; ^2^Center for Adaptive Neural Systems, School of Biological and Health Systems Engineering, Arizona State UniversityTempe, AZ, USA

**Keywords:** prosthesis, neural control, neural recordings, electrode, decoding, fascicle, peripheral nerve, simulation

## Abstract

Decoding motor intent from recorded neural signals is essential for the development of effective neural-controlled prostheses. To facilitate the development of online decoding algorithms we have developed a software platform to simulate neural motor signals recorded with peripheral nerve electrodes, such as longitudinal intrafascicular electrodes (LIFEs). The simulator uses stored motor intent signals to drive a pool of simulated motoneurons with various spike shapes, recruitment characteristics, and firing frequencies. Each electrode records a weighted sum of a subset of simulated motoneuron activity patterns. As designed, the simulator facilitates development of a suite of test scenarios that would not be possible with actual data sets because, unlike with actual recordings, in the simulator the individual contributions to the simulated composite recordings are known and can be methodically varied across a set of simulation runs. In this manner, the simulation tool is suitable for iterative development of real-time decoding algorithms prior to definitive evaluation in amputee subjects with implanted electrodes. The simulation tool was used to produce data sets that demonstrate its ability to capture some features of neural recordings that pose challenges for decoding algorithms.

## Introduction

Most commercially-available powered prostheses for upper limb amputees provide control of a single degree-of-freedom (DOF) (MotionControl, 2007). A few provide more than one DOF, but they require extensive training and exert a high demand on attention (OttoBock, 2011; TouchBionics, 2013). All of these systems fall far short of restoring the functionality of the native limb. This limitation has driven a substantial research and development effort to develop advanced powered prostheses (JHUAPL, 2014) and techniques to control the prostheses with biological signals. To afford a greater degree of control, some efforts have explored techniques to utilize signals recorded from residual or reinnervated muscle (Kuiken et al., 2009; Khokhar et al., 2010; Rehbaum et al., 2012), while others have investigated the use of signals recorded from the central nervous system (CNS) (Wolpaw et al., 1991; Zhu et al., 2005; Huang et al., 2012; Onose et al., 2012) or the peripheral nervous system (PNS) (Dhillon and Horch, 2005; Durand et al., 2008; Micera et al., 2008, 2011; Kamavuako et al., 2010; Tang et al., 2011; Wodlinger and Durand, 2011).

Although approaches that utilize recordings from muscle, CNS and/or PNS may prove to be suitable for controlling advanced prostheses, the PNS interfaces have the potential advantage of providing access to a sufficient number of signals without the risks associated with implants into the brain or spinal cord. Signals from the PNS have been recorded using various types of electrode technologies (Hoffer and Loeb, 1980; Veraart et al., 1993; Tyler and Durand, 2002). Dhillon et al. (Dhillon et al., 2004; Dhillon and Horch, 2005) demonstrated that amputees could control a one DOF robotic arm in a graded fashion using real-time decoding of signals recorded from longitudinal intrafascicular electrodes (LIFEs) implanted in the peripheral nerve stumps. These electrodes, which are fine wires that are inserted into and along a long axis of a fascicle, enable recordings from small groups of axons (up to approximately 10). Subsequent demonstrations with other electrode systems (Durand et al., 2008; Micera et al., 2008, 2011; Kamavuako et al., 2010; Tang et al., 2011; Wodlinger and Durand, 2011) further supports the investigation of PNS interfaces for prosthetic control.

For control signals derived from the PNS (as well as from muscle or from the CNS), the recorded neural signals must be transformed in order to derive the control signals to be sent to the motorized prosthesis. The transformation includes, either implicitly or explicitly, a decoding of the recorded signal to infer the intent of the user. A wide variety of algorithms to decode the biological signals for use in controlling prostheses have been developed (e.g., Wood et al., 2004; Fraser et al., 2009). The specific features of the decoding algorithm may differ depending on the type of biological signal recorded, the properties of the machine-tissue interface, and the targeted function of the prosthesis.

To evaluate the performance of a candidate decoding algorithm, the penultimate test is to use it for real-time decoding of signals recorded from an amputee as s/he performs a functional task. However, the ability to extensively utilize such a testing paradigm may be limited due to the experimental nature and limited deployment of the neuromuscular interface technologies, as well as other factors. Furthermore, such testing may not afford direct control over factors that could help to differentiate the performance of candidate decoding algorithms, such as spike overlap or the number of fibers that contribute to the signal recorded on a given electrode. Computer models of the peripheral neuromuscular system and the neural interface can be used to efficiently explore a greater range of approaches than can be readily achieved in living systems (Durand et al., 2008; Zhou et al., 2010).

In this work, we have developed a system to simulate neural recordings. This system is intended to accelerate the development and evaluation of candidate decoding algorithms by enabling the production of data sets of simulated neural recordings with known characteristics. By affording direct control over several key features of recorded neural signals, the system could be used to methodically generate data sets that could identify the advantages and disadvantages of candidate decoding algorithms.

Our simulation framework enables modeling and simulation of spinal cord motor pools and recordings by LIFEs (or other electrode technologies) from subpopulations of motor axons. The simulator can be used to produce simulated recordings from multiple electrodes for multi-DOF tasks with known motor intent, neural spike train characteristics, levels of encapsulation and signal-to-noise ratios (SNRs). This simulator was designed to facilitate direct comparison of candidate neural decoding algorithms by enabling comprehensive assessment of the effect of spike overlap, noise level, and electrode receptive field properties on algorithm performance. Here, we present a description of the model and the simulation tool as well as results of several simulations using the tool. These results demonstrate that the simulation tool can be used to systematically vary motor intent, neural firing patterns, and electrode recording characteristics in order to produce data sets that could facilitate the development and assessment of decoding algorithms for systems that use peripheral neural interfaces.

## Materials and methods

**Figure 1:**
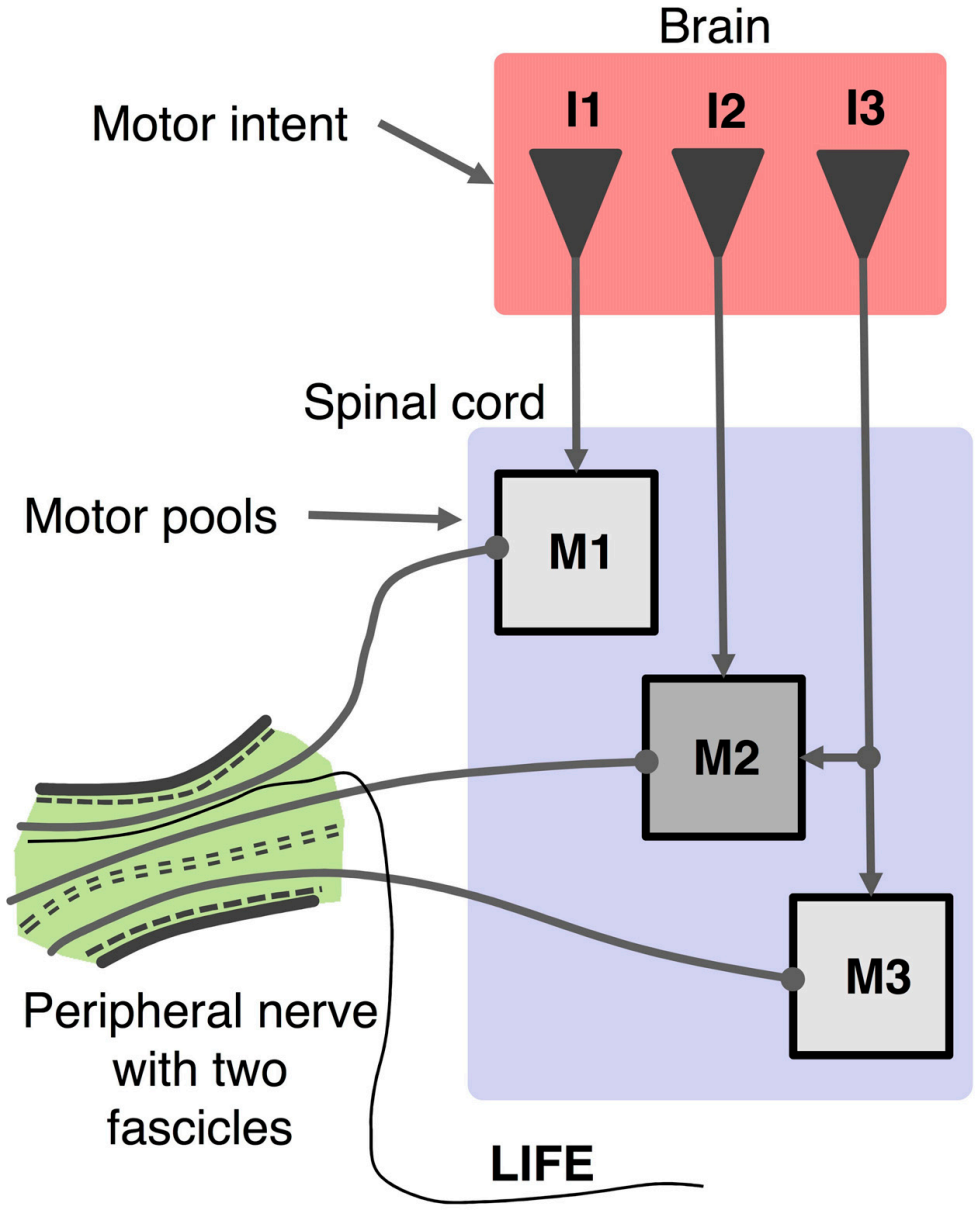
**Schematic organization of motor control system and recording of motor activity with a LIFE**. Motor intent (I: I1, I2, I3) can be represented as a multi-dimensional signal from centers in the brain to motoneurons pools in the spinal cord, which produce firing patterns in motoneurons (M: M1, M2, M3). Axons from a given motor pool tend to cluster together along the length of the peripheral nerve fascicle. The diagram shows a LIFE electrode that has been implanted into one of the fascicles of the nerve.

**Figure 2:**
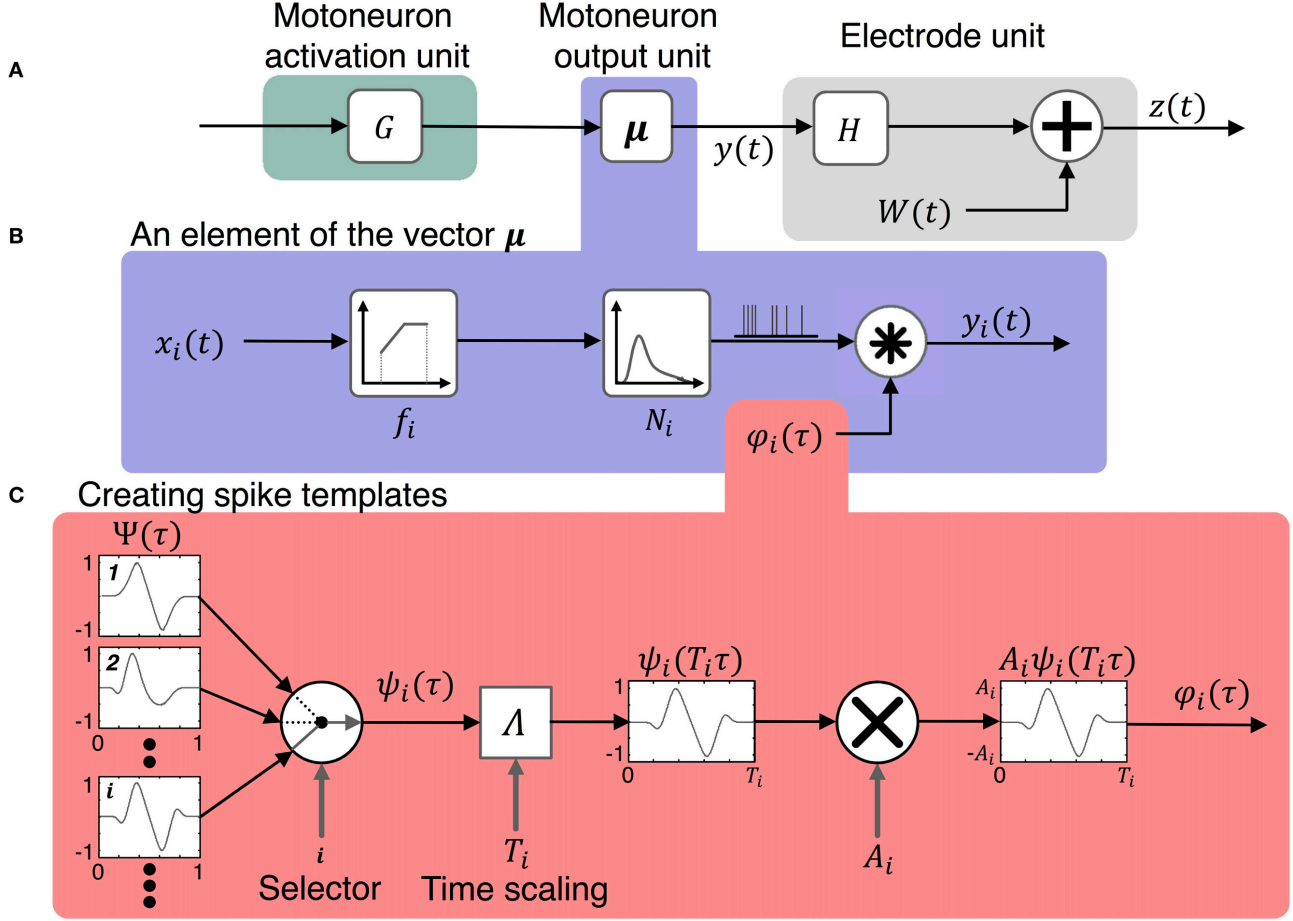
**Model for simulating the activity on peripheral nerve electrodes during motor tasks**. The model consists of three components shown in **(A)** the motoneuron activation unit, the motoneuron output unit, and the electrode unit. The input to the model is a vector of motor intent signals, ***u***(*t*), which is first transformed to activation states of motoneurons, ***x***(*t*), then to motoneuron outputs, ***y***(*t*). The motoneuron output signals combine with noise, ***W***(*t*), to produce the vector of signals, ***z***(*t*), recorded by the electrodes. The motoneuron output model includes three components shown in **(B)**: the firing rate of a motoneuron is determined by its activation state and its firing rate mapping function, the time series of pulses is the output of a point process which is then convolved with the spike template to produce the motoneuron output signals, ***y***(*t*). **(C)** Illustrates the production of spike templates with various temporal and geometric characteristics. A spike shape is selected at random from a pool of spikes of different morphologies Ψ. Then the selected spike is scaled in time by the function Λ and in amplitude by A_i_.

**Figure 3:**
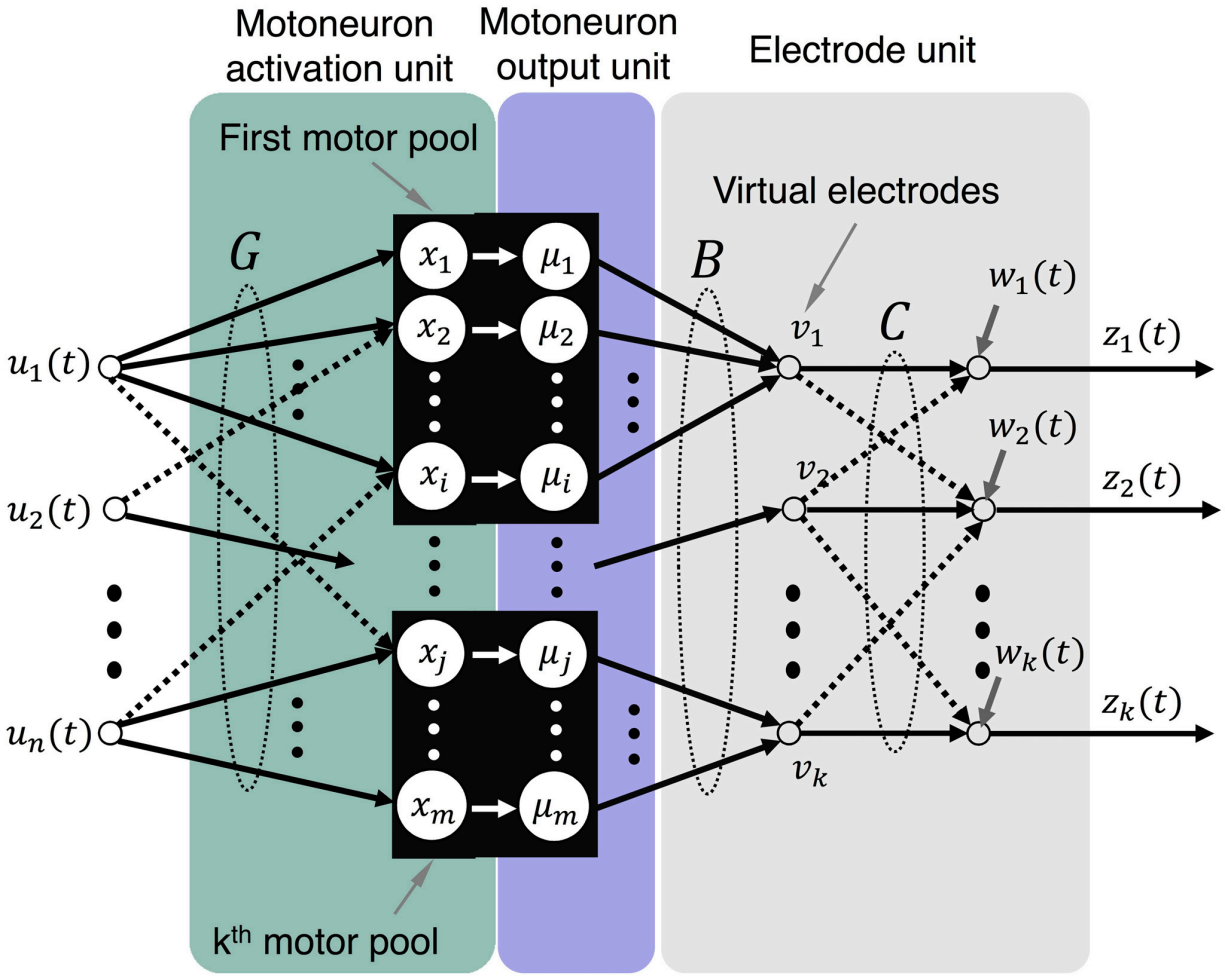
**Model representation that illustrates mappings from motor intent to electrode recordings**. The mappings being performed by each of the components are illustrated: **G** maps motor intent to motoneurons, **B** maps motoneuron outputs to virtual electrodes, and **C** maps virtual electrodes to actual electrodes.

A model and simulation system were developed to simulate the activity of motoneuron pools based on a multi-DOF input of motor intent. Figure 1 presents a schematic that represents the system that is modeled in which multiple LIFEs are implanted in peripheral nerve of an amputee to record activity of motoneurons that is driven by motor intent signals. The simulator (Figures 2, 3) consists of three primary components: the motoneuron activation unit, the motoneuron output unit and the electrode unit. Each of these is described in the sections that follow.

The simulator is implemented in MATLAB®. Simulation and user specified parameters and functions are defined using several Excel® or text documents.

### Motoneuron activation unit

The motoneuron activation unit models the transformation from motor intent to a variable that represents the membrane potential, or state, of the motoneurons.

Motor intent is the voluntary intention of a person that leads to activation of the neuromotor system to attain a motor goal (Jankowska, 1992; Carp and Wolpaw, 2010). For example, motor intent could be an attempt to flex the biceps strongly, to partially extend the wrist or to reach and grasp an object. In an amputee, motor intent may produce activity in the peripheral motor axons of the residual limb that could be recorded using a neural interface. In our simulation framework, we define motor intent as an effort to stabilize and control a single joint or coupled sets of joints. As such, realization of the motor intent would involve formulation of two essential components: an intended action and a level of effort. The intended action is the DOF to be controlled while intended effort is the intensity of that action.

The motoneuron activation unit (Figure 2A) is modeled by,

(1) x(t)=Gu(t),

where ***u***(*t*) is an *n* × 1 vector, where *n* is the number of motor intent signals. This is a vector quantity in which the individual components represent motor intent normalized by maximum intended effort. ***G*** maps the motor intent signals to motoneurons. It is an *m* × *n* matrix, with *m* ≥ *n*, where *m* represents the number of spinal cord motoneurons in a motor pool. ***x***(*t*) is the *m* × 1 vector of the motoneuron activation states. Here, the activation state, *x_i_*(*t*), represents the membrane potential of motoneuron *i* at the site of action potential initiation (axon hillock). This represents the time-varying state that will determine the instantaneous firing rate of a motoneuron. Note that the motor intent vector represents direct inputs to motor pools through the connectivity matrix, ***G***. Uniform values in a row of ***G*** would be used to simulate uniformity of inputs across the set of the motoneurons (Fuglevand et al., 1993); variations in these values would simulate a situation in which some motoneurons in the pool received stronger input than others. Indirect motor pathways are not included in the current implementation of the simulator and motor intent signals are modeled as graded values, not firing patterns.

In the simulator, the motor intent vector ***u***(*t*) Equation (1) is a set of independent functions over a time interval [0, *T*] that is specified by the user prior to the start of the simulation. Users have the option to set each component of the vector ***u***(*t*). For example, motor intent can be set as a square wave, ramp-and-hold, sinusoid, etc. Alternatively, a dynamic model can be used to generate motor intent signals for a task such as reaching. The structure and values of ***G*** are specified in a parameter file.

### Motoneuron output unit

This component of the model (Figure 2A) represents the transformation from motoneuron state (time-varying membrane potential just prior to the axon hillock) to time-varying extracellular potential just outside the axon. This unit is responsible for generating spike events based on the state of the motoneurons and producing the extracellular voltage waveform based on the spike events.

Alpha motoneurons, which comprise the motor pool, fall into three subclasses according to the contractile properties of the muscle fibers they innervate: fast-twitch fatigable (FF), fast-twitch fatigue-resistant (FR), and slow-twitch fatigue-resistant (S). These three fiber types differ in size (of the muscle fiber and the motoneuron) recruitment characteristics, and range of firing rates. The recruitment of motoneurons in a motor pool is postulated to follow the size principle (Henneman and Mendell, 2011)—small motoneurons fire first and as motor drive increases, larger motoneurons are recruited and contraction strength increases. Variations in excitability of motoneurons within the pool may be the primary mechanism for this orderly recruitment (Fuglevand et al., 1993). Small motoneurons connect to slow fibers while larger ones innervate fast twitch fibers (Brown et al., 2006; Carp and Wolpaw, 2010). The firing rates observed in slow fibers are lower than the rates observed in large fibers (Cisi and Kohn, 2008).

The motoneuron output unit (Figures 2A,B) is modeled by

(2) y(t)=μ(x(t))

where ***y***(*t*) is an *m* × 1 vector that represents the extracellular potentials at each axon and μ is a function that maps the activation states, ***x***(*t*), of the motoneurons to ***y***(*t*).

The motoneuron output model includes three components: the first component determines the mean firing rate based on activation state and motoneuron properties; the second produces a train of spike events based on mean firing rate and the specification of a point process function for spike event timing; the third produces the time series of the extracellular potential based on the spike event timings and the spike template. Each of these components of the model is described in more detail below.

**Figure 4:**
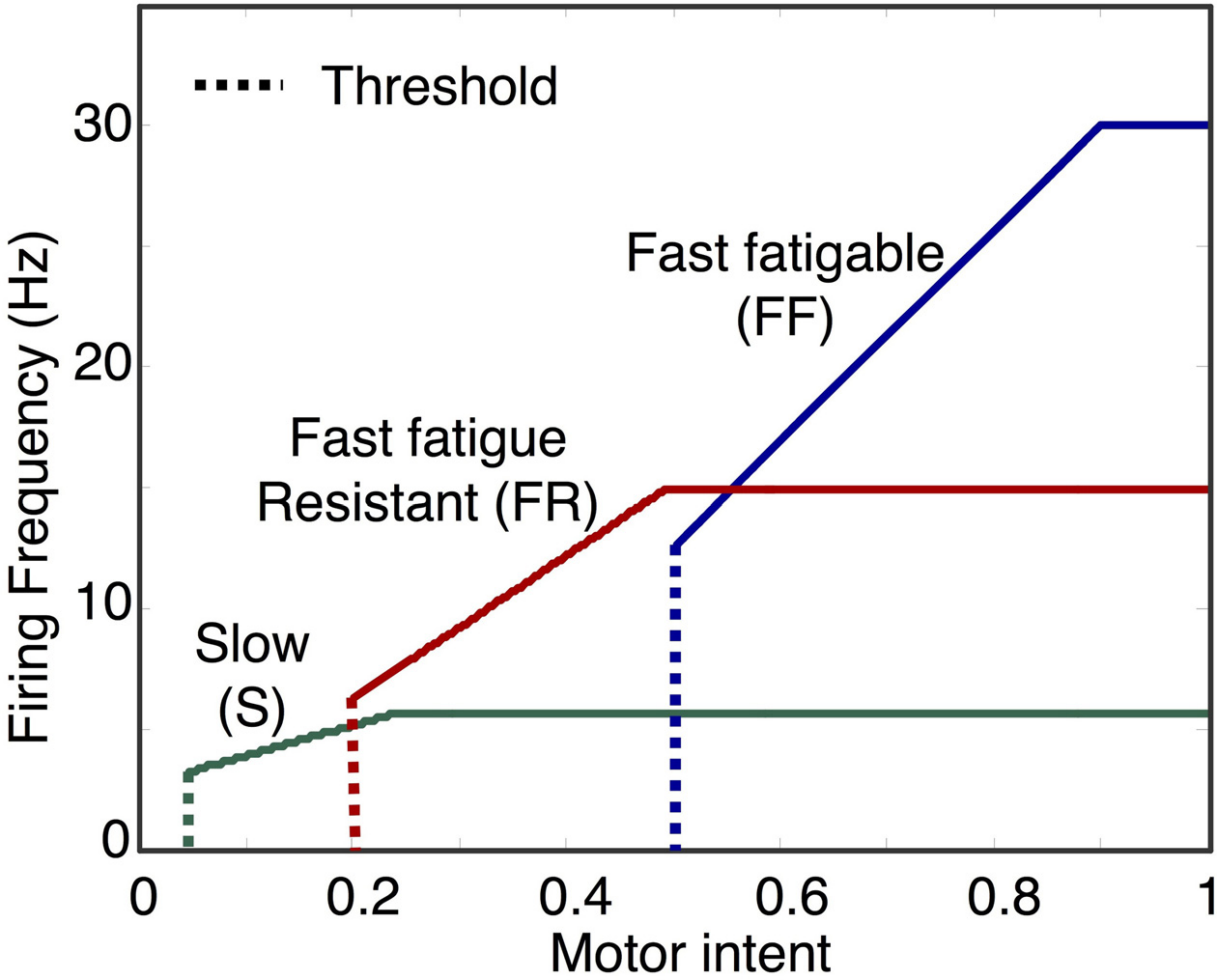
**Examples of motoneuron input/output curves**. The first component of the motoneuron output unit determines motoneuron firing rate based on the activation state of the motoneuron. In the model, this is implemented using a piecewise linear function with threshold and saturation. The plot shows examples of curves representing the mapping from motor intent to firing frequency for three motoneuron pools, one for each of the three fiber types. Note that the mapping values specified in this example will produce sequential recruitment of the slow (S), fast fatigue resistant (FR), and fast fatigable (FF) as motor intent is increased.

The mapping of motor unit activation to firing rate of the various types of motoneurons are represented schematically in Figure 4. For each motoneuron, the firing rate is given by

(3) $$f(x)=\left\{ \begin{array}{l} 0,\;0\leq x<x_{thr},\\ \kappa_{f}x,x_{thr}\leq x<x_{sat},\\ f_{sat},x\geq x_{sat}, \end{array}\right.$$

where the slope κ_*f*_ of the input/output response curve is given by,

(4) $$\kappa_{f}=\frac{f_{sat}-f_{thr}}{x_{sat}-x_{thr}}$$

where f is the frequency of firing in Hz. *x_thr_* is the activation state above which a motoneuron begins to fire. *f_thr_* and *f_sat_* are the minimum and maximum frequency of firing for a motoneuron, while *x_sat_* is the activation level at which a the firing rate saturates. The activation state *x* is normalized between 0 and 1, where 1 represents maximum activation. *x_thr_* determines the effort at which a particular motoneuron is recruited. In the simulator, *x_thr_*, *f_thr_*, *x_sat_*, and *f_sat_* can be set by the user for each motoneuron and can therefore be used to specify the input/output properties of a motor pool.

The firing rate, f, represents the time varying mean instantaneous firing rate, but the actual spike timing is produced by one of several point process functions (Fuglevand et al., 1993; Cisi and Kohn, 2008; Zhou et al., 2010) described below. Let

(5) $$N\left(\xi \right)\sim \{\begin{array}{c} \xi \\ Poisson\left(\xi \right)\\ TruncatedGaussian\left(\xi ,\sigma \right)\\ Gamma\left(\xi ,\sigma \right)\\ Uniform\left(\xi ,w\right) \end{array}$$

be a stochastic point process having one of the distributions listed above. The activation state x determines the mean interspike interval (ISI, ξ = 1/*f*). The simulator provides the option of selecting one of the different point processes for spike trains: Identity, Poisson, Truncated-Gaussian, Gamma, or Uniform Equation (5). The Identity process produces a regular spike train for testing other simulator functionalities. The Poisson process produces an irregular spike train where the variability is dependent on the mean firing rate. The Gaussian distribution has been selected for use in prior modeling studies (Fuglevand et al., 1993) based on some reports of firing rate variability. In the last three processes, the variability in ISI can be set to be independent of the mean ISI. This is useful for evaluating the performance of decoding algorithms under different levels of ISI variability while the mean ISI remains fixed.

The third component of the motoneuron output model produces the time series of the extracellular potential based on the spike event timings and the spike template. Each motoneuron output spike has a characteristic morphology, amplitude and duration. The shape of the extracellular spike is influenced by the size of the axon, the number and type of voltage gated channels, whether or not it is myelinated, and the general health of axons, since atrophy after amputation can alter spike shape (Dhillon et al., 2004).

Extrinsic factors that influence the shape of spike recorded from an extracellular electrode are the recording electrode material type, geometry, location, and orientation with respect to neural sources as well as characteristics of the tissue-electrode interface such as the degree and type of encapsulation. To simplify the real-time simulation process, we have chosen to include the effects of electrode type in the shape of the spike templates. Therefore, the spike template represents the shape and duration of the extracellular effect of the axonal spike train as viewed by an electrode. Note that the effect of electrode location and other extrinsic factors that affect amplitude are represented in the electrode unit. This structure streamlines the simulation process by incorporating all of the temporal aspects of a spike in one template; all other processes that affect the recorded signals involve only addition (superposition) and multiplication (scaling).

Extracellular waveforms occupy a frequency bandwidth between 100 Hz and 10 kHz depending on the recording electrode (Horch and Dhillon, 2004; Plonsey et al., 2007; Gosselin, 2011). Some examples of shapes of action potentials recorded using LIFEs have been provided in the literature (Malagodi et al., 1989; Lefurge et al., 1991; Lawrence et al., 2004; Dhillon and Horch, 2005; Micera et al., 2008).

**Figure 5:**
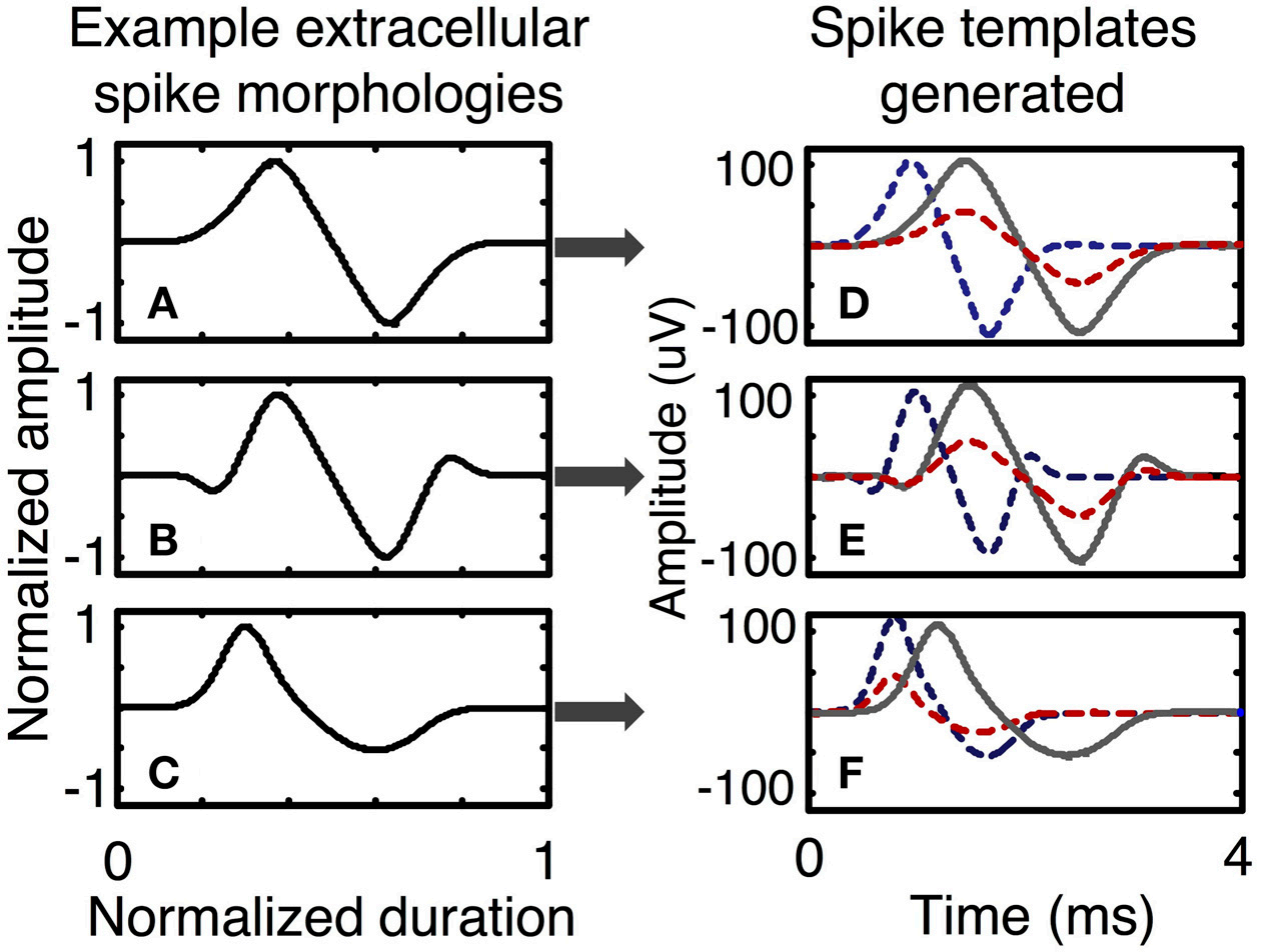
**Examples of spike templates**. Three spike morphologies with normalized amplitudes between (− 1, 1) and normalized duration between (0, 1) are scaled in time and amplitude to form a multitude of spike templates. A spike template is a characteristic of a neuron. Spike morphologies are classified in terms symmetry and the number of peaks and troughs. Plots **(A–C)** present spike morphologies that are: symmetric with one peak and one trough, symmetric with two peaks and two troughs, and asymmetric with one peak and one trough. Other spike morphologies are possible and can be directly programmed in the simulator. After scaling in amplitude and time, each spike morphology can be used to generate several spike templates, as shown in plots **(D–F)**, each of which has three spike templates generated from one spike morphology.

In the simulator, spike shapes are specified by the user in a process that includes several steps. First, the user selects normalized spike morphologies (Figure 5). Spike morphologies are generated by differentiating Gaussian and Gamma functions, which can produce a variety of spike wavelets similar to spike shapes reported in the literature. The spike wavelets are normalized in amplitudes between (−1, 1) and normalized in duration between (0, 1). The spike-morphologies are then scaled in amplitude and duration by the simulator using parameters that can be specified by the user (Figure 2C).

Let **Ψ**(*t*) be a *m* × 1 vector function that encodes spike shapes of a motoneuron. Each component of Ψ, ψ_*i*_(*t*), will have the following properties,

(6) $$\int_{-\infty }^{\infty }\psi_{i}\left(s\right)ds=0,$$

and

(7) $$\int_{-\infty }^{\infty }\psi_{i}{}^{2}\left(s\right)ds<\infty .$$

Now, we can define output of a motoneuron *i* as follows (Figure 2):

(8) $$\mu_{i}\left(x_{i}(t)\right)=\int_{0}^{t}\psi_{i}(t-\tau )dN\left(f_{i}(x_{i}(\tau ))\right).$$

If *N* is a Poisson process, then we can rewrite the function μ_*i*_ as

(9) $$\mu_{i}\left(x(t)\right)=\sum_{j\;=\;0}^{\infty }\int_{0}^{t}\psi_{i}(t-\tau )\delta (\tau -\tau_{j})d\tau .$$

where δ (τ − τ_*j*_) is the delta function and τ_*j*_ is spike event time, a function of the input/output response map *f_i_* and Poisson process.

To implement the motoneuron output units in the simulator, for each motoneuron the user specifies an input/ouput response curve, a firing model (e.g., Poisson, Gaussian) and a spike template. Spike templates are generated by a subunit of the simulator (Figures 2C, 5).

### Electrode unit

The output of the electrode unit is the summation of signals from the motoneurons in the vicinity of the electrode. The number of units and their relative contributions will depend upon the design of the electrode, its location in or near the fascicle, and the properties of the tissue between the motor axon and the electrode. The model of the electrode unit is designed to represent each of these factors, which are described below.

#### Characteristics of LIFEs

In this study, we have implemented a model of the LIFE electrode. In studies that performed peripheral nerve recordings in animal models, LIFEs have been fabricated from 25, 50, or 100 μm diameter insulated 90%Pt–10%Ir. A 1 mm recording site is made by removing part of the insulation (Malagodi et al., 1989; Lefurge et al., 1991). Each LIFE is placed in a fascicle so that it is aligned with the axons.

Superposition is the summation of neural signals from multiple sources on a single recording electrode. The amount of superposition depends on the structure and relative position of the electrode with respect to the neural sources. A LIFE with these dimensions and placement typically records from 6 to 10 axons (Lefurge et al., 1991). The amplitude of the component from each axon will depend upon the strength of the signal and the distance of that axon from the electrode.

Crosstalk occurs when a neural electrode picks up neural signals from motor axons emanating from different motor pools. This may lead to superposition of two or more intended motor actions on a single electrode recording. However, it has been reported that peripheral nerves are somatotopically organized even at fascicular and subfascicular level (Hallin, 1990). Given this organization, an electrode that records from a small number of fibers is likely to record primarily from motor axons derived from the same, or related motor pool (Topp and Boyd, 2012).

Spike overlap refers to the temporal coincidence of two spikes from different motor axons on one electrode. The overlap of spikes from two or more waveforms could be constructive, which would result in one large spike, or destructive, which would result in a small amplitude spike. Either of these could distort spike shapes, lead to a failure to in spike detection, and alter the apparent firing frequencies in recorded neural activity.

A system of multiple LIFEs implanted in multiple peripheral nerve fascicles could record from multiple motor pools and reflect different motor actions. The knowledge of nerve gross anatomy helps guide the placement of electrodes into nerves that carry information related to the targeted motor actions, but it is not currently possible to surgically target specific regions within a fascicle of a nerve or motoneurons from a specific muscle. The relationship between motor intent and the signal recorded on each electrode must be determined (decoded) experimentally. Similar decoding procedures have been carried out for cortical and other peripheral interfaces (Allison et al., 1992; Donoghue, 2002; Dhillon et al., 2004; Dhillon and Horch, 2005; Velliste et al., 2008; Blakely et al., 2009; Halder et al., 2011; Krusienski and Shih, 2011; Hochberg et al., 2012).

Drift is unwanted relative motion between the neural interface and neural sources. Drift can affect the recorded firing patterns and crosstalk. Any increase in the distance between the axon and the electrode would attenuate its contribution to the recorded signal.

Encapsulation is the accumulation of biological matter on the neural interface as a result of the tissue response to the electrode (Lefurge et al., 1991; Polikov et al., 2005). Encapsulation attenuates neural signals and can lead to dysfunctional electrodes.

The noise in recordings from LIFEs (or other electrodes) emanates from a number of sources: activity of muscles in the vicinity of the electrode, electrocardiac signals, background neural activity from motor or sensory axons, tissue thermal noise, thermal and impedance properties of the neural interface, the recording system and the recording environment (e.g., power hum and flicker noise).

#### Electrode unit: model

In the simulator, a mapping matrix is used to direct signals from one or more motor axons to each LIFE (Figures 2A, 3). The value of each element in the matrix represents the sum of the effects of distance from the axon to the electrode, drift, and encapsulation. Noise is incorporated as additive signal.

The neural component of the signal recorded on each electrode is a weighted sum of the extracellular signals generated by the motoneurons (Figure 2A) and is described by

(10) z(t)=H(y(t))+W(t)

where **z**(*t*) is a vector representing the signals recorded on each of *l* electrodes, ***y***(*t*) is the vector representing the activity of *m* motor axons, ***H*** is a *l*× *m* matrix that maps motor axon activity to electrodes and ***W***(*t*), which represents noise, is an *l* × 1 vector. The values for ***H*** reflect the location of the electrodes with respect to the motor axon. For example, a small value for an element of ***H*** would indicate an axon that is distant from the electrode and would therefore contribute weakly to the recorded signals.

***H*** can be configured by the user to test different electrode configurations and recording scenarios. For example, the recording from a LIFE electrode may include substantial contributions from 6 to 10 motor axons signals, the recording from an electrode on a Utah array may include substantial contributions from 1 to 6 motor axons. To simulate recordings from fibers that are close to an electrode with a low degree of encapsulation, the elements of ***H*** should be set to high values (close to 1); the effect of increased distance or encapsulation can be simulated with lower values to achieve signal attenuation.

***H*** can be defined as the product of two matrices:

(11) H=CB

where ***B*** is a *l* × *m* matrix, ***C*** is an *l* × *l* matrix, *m* is the number of motor axons, *l* is the number of electrodes. The matrix ***B*** maps activity from a subset of related motor axons (i.e., the same motor pool) into activity on a set of virtual electrodes ***v***(*t*) (Figure 3).

(12) v(t)=B(y(t)).

In this formulation signals detected by the virtual electrodes represent pure motor commands destined to a particular muscle. The mapping matrix ***C*** is the degree of crosstalk between motor pools or, in this case, virtual electrodes. This representation enables explicit specification of crosstalk that is separate from the specification of the mapping to virtual electrodes.

Since LIFEs record from a small number of fibers that are likely to be in the same motor pool, we assume that ***C*** is nearly the Identity matrix. That is, cross-talk between motor pools is negligible. In this case, the LIFE's electrode signal **z**(*t*) is given by

(13) z(t)=Bv(t)+W(t).

In Equation (10), ***W*** is the sum of all noise sources in the environment. In the simulator, noise is modeled as power-law noise (i.e., 1/*f*^β^) whose amplitude and β parameter can be specified by the user. Alternatively, the user can specify band-limited Gaussian white noise and specify the SNR or provide an additive noise time series using an input file. In this case, the standard deviation of the noise will be determined by

(14) $$\sigma_{noise}=\frac{Q_{99.9}-Q_{0.1}}{3\;SNR},$$

where *Q*_99.9_ and *Q*_0.1_ are the 99.9% percentile and 0.1% percentile of the pure neural signal recorded by the electrode.

### Operation of the simulator

In order to implement a simulation run, the user must specify the following simulation parameters:

1. Input/output response curves for each motoneuron, including threshold motor intent and initial firing frequency and saturation motor intent and firing frequency. Note that recruitment characteristics are indirectly specified by the threshold motor intent and saturation point.
2. Spike template for each motoneuron including: spike shape, duration, and amplitude.
3. Firing model—Poisson, Gaussian, etc.
4. Motor intent to motoneuron mapping matrix, ***G***.
5. Motoneuron to electrode mapping matrix, ***H***.
6. Noise model including SNR ratio and bandwidth.

### Demonstration of simulator capabilities

The simulator was used on specific models in various scenarios to demonstrate its capabilities with a particular emphasis on producing data sets with characteristics that could pose challenges for neural decoding algorithms, such as: recordings from multiple axons with different spike morphologies and spike train characteristics (Simulation run **1**), recordings produced by motor intent commands with more than one DOF (Simulation run **2**), recordings produced by motor intent commands at slowly varying or different levels of quasi steady-state activity (Simulation run **3**), and recordings with substantial spike overlap (Simulation run **4**).

Simulation run **1** was set up to demonstrate the different spike trains from fast and slow motor units and to demonstrate superposition of signals from different motor pools. This model included 5 electrodes in the vicinity of S and FF motor units with a ramp in motor intent (1 DOF). Six motor units of each type (S and FF) were simulated; the spike morphology used for the contribution of each motor unit was the shape shown in Figure 5A. The parameters for each motor unit were selected from a uniform random distribution across a pre-specified range. The ranges of values used for spike duration was 4–6 ms for S, 2–4 ms for FF; the ranges for spike amplitudes were for 45–65 for S, 95–105 for FF; the ranges for firing frequencies at threshold were 1–5 Hz for S and 12–19 Hz for FF; ranges for firing frequencies at saturation were 16–18 Hz for S, 25–30 Hz for FF; ranges for motor intent threshold were 0–10% for S, 35–65% for FF; ranges for motor intent saturation were 40–50% for S, 80–100% for FF. The Poisson model for spike timing variability was used. The weights on contributions of the neurons to the recorded signal (values in the ***H*** matrix) were assigned amplitudes that were equally spaced over the range from 0.5 to 1 and each electrode had additive noise with SNR = 3 (average across the set electrodes). These simulations demonstrate recordings from electrodes that record from 1 S unit, 6 S units, 1 FF unit, 6 FF units, and 3 S and 3 FF units, respectively.

Simulation run **2** was set up to demonstrate multiple DOF motor intent and a composite of two motor pools onto one electrode. This model included 3 electrodes and a 2 DOF motor intent signal: one electrode was modeled to be in the motor pool of the first motor intent signal; another electrode was modeled to be in the motor pool of the second motor intent signal; the third electrode was modeled to be in the vicinity of both motor pools. The parameters used for this run were the same as the mixed fiber electrode (3 S and 3 FF) in simulation run 1 except that for the third electrode the cross-talk matrix was set to equally weight inputs from the two motor intent signals (0.5 for all matrix elements).

Simulation run **3** was set up to demonstrate the effect of motor intent commands at slowly varying or different levels of quasi steady-state activity. These simulated examples are also used to demonstrate the qualitative similarity of the simulated traces to recordings from the peripheral nerve of an amputee. This model included a single DOF motor intent and additive noise; the parameters of the model were specified to approximate the characteristics of actual recordings from the peripheral nerve of an amputee (Dhillon et al., 2004; Dhillon and Horch, 2005). The first recording was from a trial in which an amputee was requested to produce a ramp in motor intent. To produce the simulated data set, we configured the electrode to record from 6 motor axons, since experimental data has indicted that a LIFE with these dimensions and placement typically records from 6 to 10 axons (Lefurge et al., 1991). Additionally, the SNR ratio in the simulator was set to be equal to the SNR calculated from neural data. Motor intent was estimated from the real neural data using a simple moving average decoder (i.e., the time series was low pass filtered using a 200 ms moving average window). Then, we produced a simulated motor intent signal that closely resembled the extracted motor intent in time and amplitude but free of noise and irregularities. We used this motor intent signal to generate the simulated neural data using the specified set of neuron characteristics and electrode characteristics.

The second group of simulations in this run was set up to mimic a sequence of 3 trials in which an amputee was requested to produce a steady-state value in motor intent at a low, moderate and then high level, respectively. This model used a set of 30 electrodes with varying number of axons (2, 4, 6, 8, or 10) and varying properties of the motor pool (all S, all FF, or an equal mix of S & FF). Each was simulated under three conditions (motor intent levels of low, moderate, and high steady-state values). For each steady-state trial, the power spectrum was calculated from the bandpass-filtered (4th order; 80 Hz–4 kHz) time-series data using the Welch method (0.5 s window; 50% overlap) and the total power, mean frequency and estimated motor intent for each trial were calculated. In all trials in this run, neural recording amplitudes of both simulated and experimental data were scaled using the standard deviation of the quiescent phase (i.e., a null motor intent) on that electrode.

Simulation run **4** uses a large set of simulation runs that was designed to demonstrate the effect of firing rates and the number of axons per electrode on spike overlap. This model used a set of 15 electrodes with varying number of axons (2, 4, 6, 8, or 10) and varying properties of the motor pool (all S, all FF, or an equal mix of S & FF). Each was simulated under 10 conditions (motor intent levels of up to 100% in increments of 10%). For each simulation run, the composite firing rate (total number of spikes from the set of neurons contributing to the electrode) and the % spike overlap (the percentage of the time in the simulation run where a spike was present on more than one axon contributing to that electrode) were calculated.

## Results

**Figure 6:**
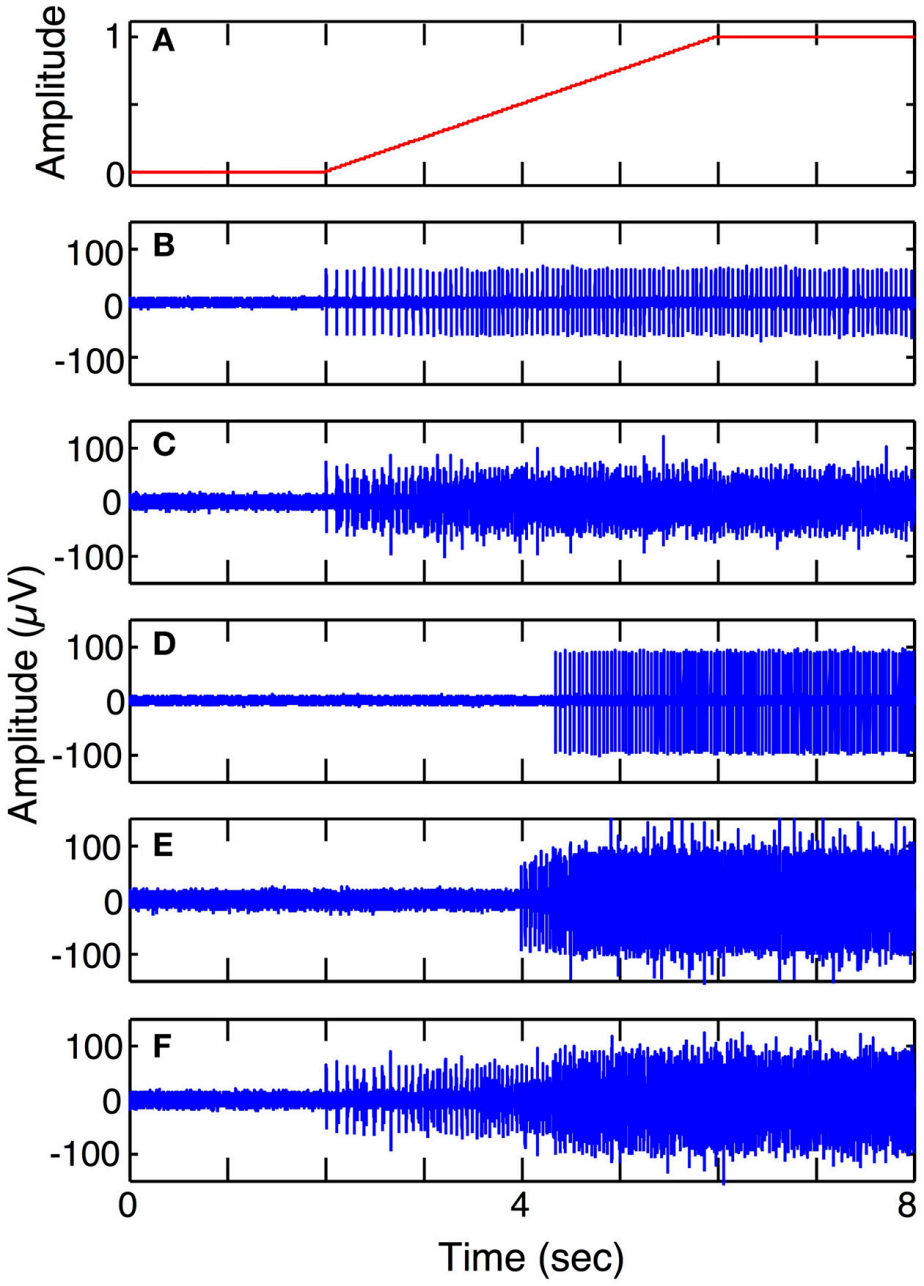
**Simulated LIFE recordings from FF and S motor axons (Simulation run 1)**. Plot **(A)** shows motor intent, which in this case is a slow ramp to maximum. Plot **(B)** shows firings of one S motor axon. Plot **(C)** shows a LIFE recording from six S motor axons. Plot **(D)** shows firing of one FF motor axon. Plot **(E)** shows a LIFE recording from six FF motor axons. Plot **(F)** shows a LIFE recording from a mixture of three S and three FF motor fibers. Each simulated recording includes additive Gaussian noise with SNR = 3.

Figure 6 shows simulated LIFE recordings from fast and slow motoneurons in response to a slow ramp and hold motor intent (Simulation run **1**). Each motor axon contributes different firing patterns to a LIFE electrode recording. S fibers have sparse firing, longer spike duration and smaller amplitudes while FF fibers have larger amplitudes shorter spikes and more dense firing patterns. A LIFE electrode, depending where it is placed in a nerve fascicle, could either record activity from S, FR, FF or a mix of motor axons. In this simulation, the motor intent signal was a ramp up to a maximum contraction (Figure 6A). Figure 6B shows action potentials from a single S motor axon and Figure 6C shows a recording from the LIFE that is the superposition of signals from six S motor axons. Figure 6D shows firings of a single FF motor axon and Figure 6E shows a recording from the LIFE that is the superposition of signals from six axons of FF motoneurons. Figure 6F is a LIFE recording from a set of three S and three FF motoneurons. These plots demonstrate that the properties of the motoneurons as specified for the FF and S fibers produce different contributions to the LIFE recording and demonstrate the superposition of signals from many motoneurons onto a single LIFE recording.

**Figure 7:**
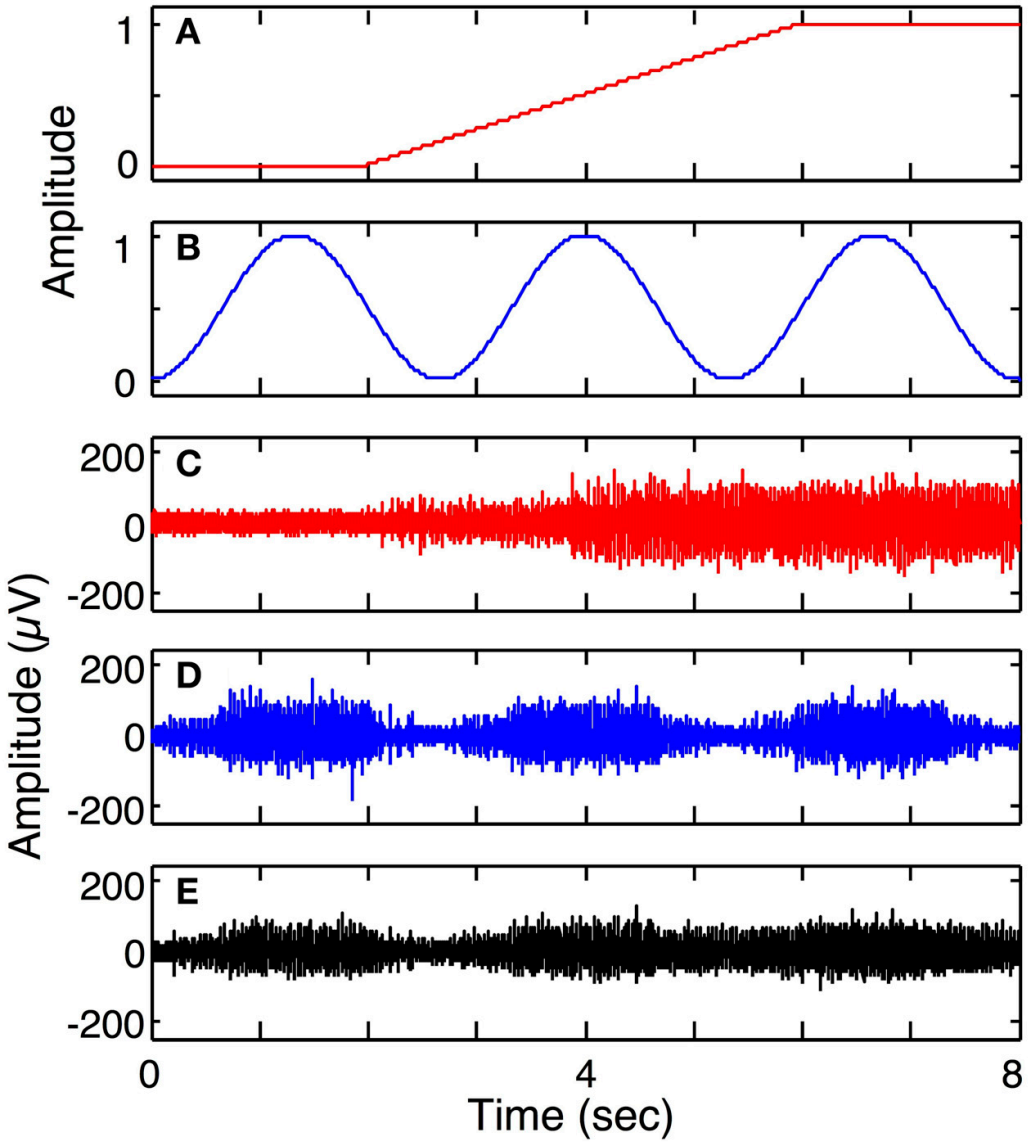
**Simulated recording from a 2 degree of freedom (DOF) task (Simulation run 2)**. Plot **(A)** shows motor intent pertaining to the 1st DOF, for example, performing a grip and hold; plot **(B)** shows motor intent pertaining to 2nd DOF with a series of contractions and relaxations. Plot **(C)** shows recording from a LIFE electrode recording from motor axons associated with the first DOF, while plot (**D)** shows a LIFE recording associated with the second DOF. Plot **(E)** shows a recording from a LIFE electrode picking up signals from the two motor pools associated with the first and second DOFs.

Figure 7 demonstrates the ability of the simulator to generate simulated LIFE recordings for a multiple-DOF task (Simulation run **2**). The motor intent signals were independently specified to represent a ramp-and-hold for the first DOF (Figure 7A) and a series of contractions and relaxations for the second DOF (Figure 7B). Each of these motor intent signals produced activation in a motor pool. Three electrodes were placed such that the first recorded signals from the first motor pool (Figure 7C); the second electrode recorded from the second motor pool (Figure 7D); and the third recorded signals from both motor pools (Figure 7E).

**Figure 8:**
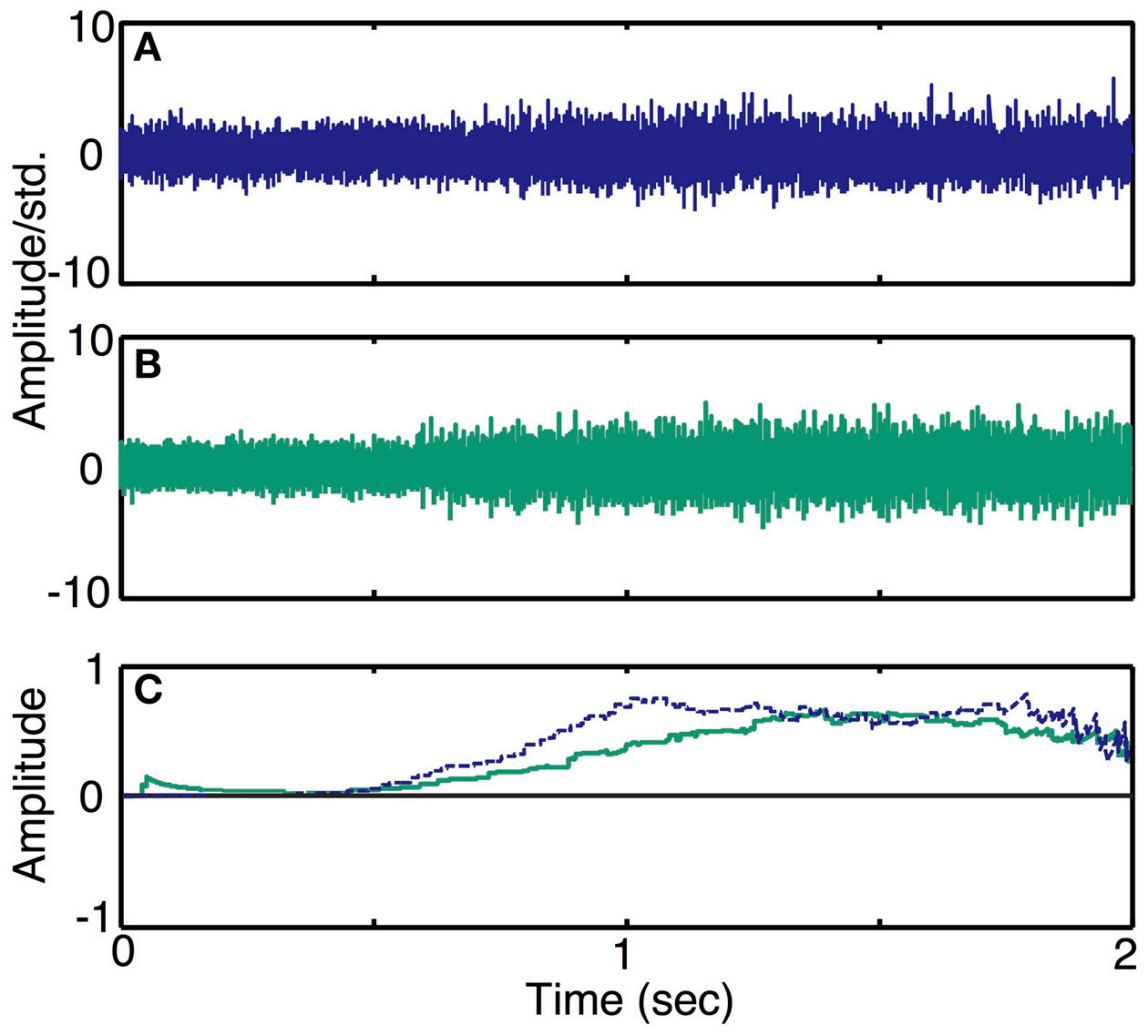
**Simulated recordings from slowly varying commands in motor intent and comparison with data recorded using LIFE electrodes in an amputee (Simulation run 3)**. Experimental data from a ramp and hold task (Dhillon et al., 2004) is plotted in **(A)**. A simulated recording from a ramp and hold task is plotted in **(B)**. Both simulated and experimental data were scaled using the standard deviation of the quiescent phase (i.e., a null motor intent). Plot **(C)** shows a plot of decoded motor intent: the blue trace is from the actual LIFE recording in **(A)**, the green trace is from the simulated data shown in **(B)**.

To demonstrate the ability of the simulator to produce neural recordings that can mimic actual neural recordings (Simulation run **3**), we compared simulated traces to data acquired by a LIFE implanted in an amputee (Dhillon et al., 2004; Dhillon and Horch, 2005). Figure 8 demonstrates that the simulated ramp data (Figure 8B) is qualitatively similar to the actual data (Figure 8A). In addition, a moving-window sign-test (200 ms) was used to compare the squares of simulated and experimental data. This analysis indicated that the experimental and simulated data are not significantly different from each other (*p* ≈ 1). Figure 8C shows the decoded motor intent signals from simulated and real data. Note that this comparison between simulated and real data is limited by the nature of the recorded neural data, because we did not have an independent measure of motor intent. Thus, the motor intent signal used to generate the simulated neural recording is the result of the simplified decoding scheme and is not a true representation of the original motor intent signal.

**Figure 9:**
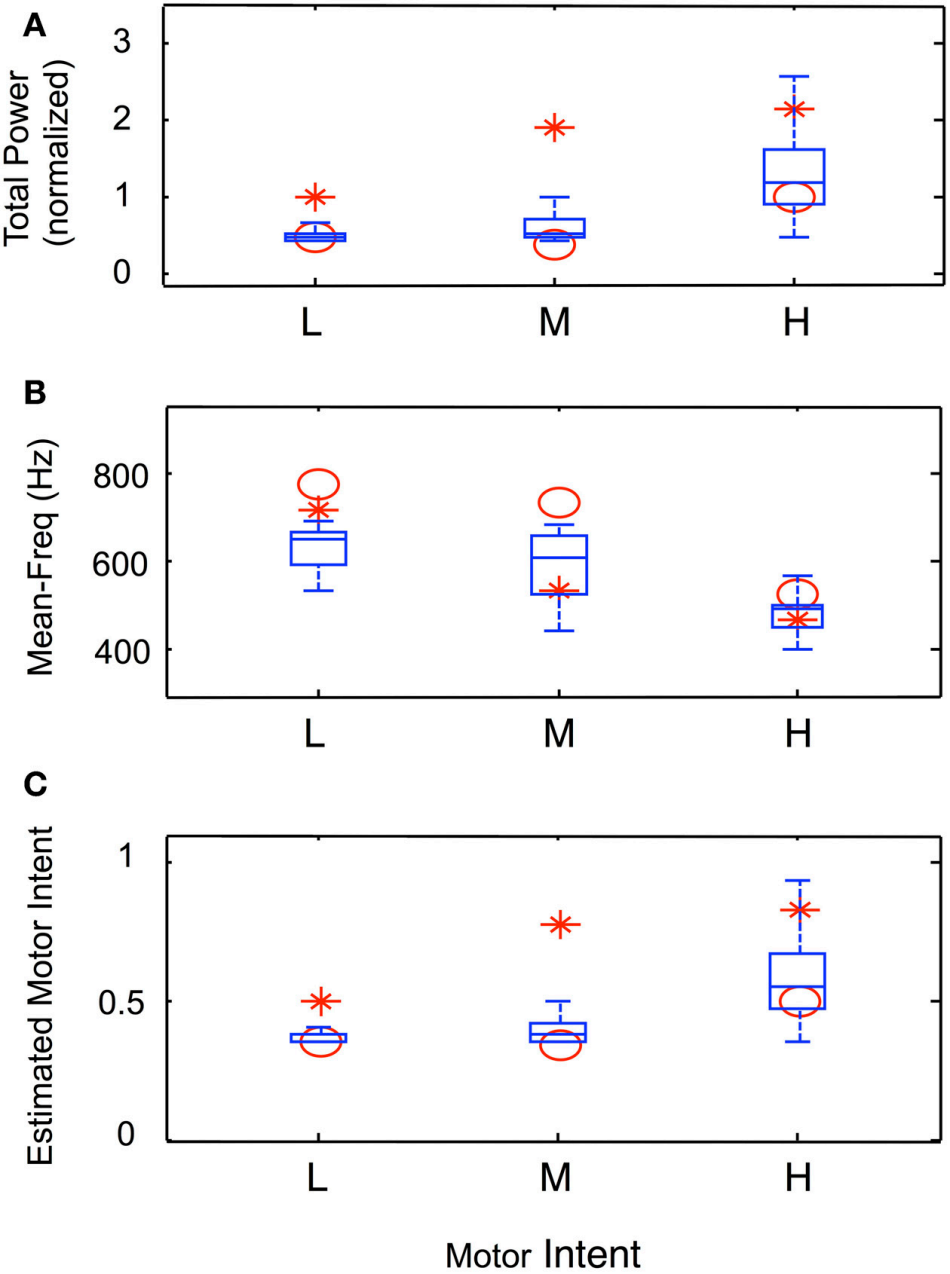
**Characteristics of simulated steady-state contractions at different levels of motor intent and comparison with data recorded using LIFE electrodes in an amputee (Simulation run 3)**. Normalized total power **(A)** and mean frequency **(B)** calculated from the power spectra from simulated and experimental data. The box-and-whisker plots at each level of motor intent present the mean, quartiles and 99-percentile ranges of data from 30 simulated electrodes. The calculated values for total power and mean frequency from the spectra of experimental data from two amputee subjects are superimposed (red symbols). Similarly, the estimated motor intent values from simulated and experimental trials are presented in **(C)**.

Data from trials in which an amputee was asked to produce steady-state levels of motor intent are summarized in Figure 9. Figures 9A–C present the normalized total power, mean frequency and estimated motor intent values for each motor intent level. The values calculated for the 30 electrodes used in the simulation run are presented in the form of box-and-whisker plots (quartiles and 99 percentile ranges); the data calculated from trials at each motor intent level from two amputee subjects are superimposed. These data indicate that, for both the simulated and actual recordings, as the level of motor intent increased from low to medium to high, total power increased, the mean frequency decreased, and the estimated motor intent value increased.

**Figure 10:**
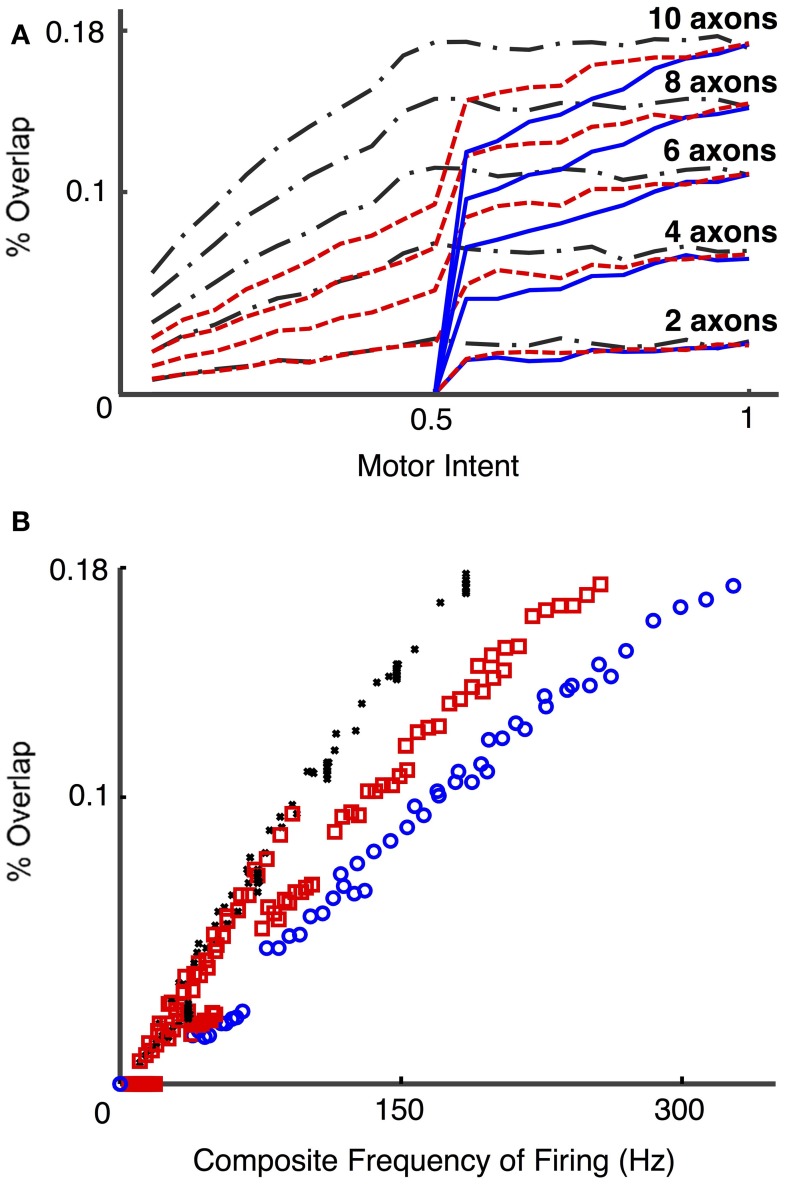
**Percent overlap as a function of motor intent and spike frequency (Simulation run 4)**. These plots present data from a set of simulations using different motoneurons pools (S, FF, and mixed S & FF) that provide signals to a set of LIFEs. S motoneurons had spike durations of 4 ms and had firing frequencies that ranged from 5 to 18 Hz over the lower half of the motor intent range; FF motoneurons had spike durations of 2 ms and had firing frequencies that ranged from 18 to 35 Hz over the upper half of the motor intent range. 15 electrodes were simulated with different combinations of fiber type (all S, all FF, or a mix of S & FF) and number of neurons contributing (2, 4, 6, 8, 10). Percent overlap represents the percentage of the recording time in which there was overlap of 2 or more spikes. Composite frequency was calculated as the total number of spikes summed across all neurons that contribute to a particular electrode. Plot **(A)** shows the percent overlap on recordings from LIFE electrodes as a function of motor intent. Note that percent overlap is higher for electrodes that record from more neurons and that it increases as a function of motor intent. Plot **(B)** presents results from the same set of simulations, but with the data plotted as a function of composite frequency. On the plots, the black, red, and blue lines/markers indicate values derived from electrodes that record from S, mixed and FF motoneurons, respectively. Note that with this specification of motoneurons (spike duration and rates), the highest value for percent overlap is less than 20% and that electrodes that record signals from S motoneurons have higher values of spike overlap for a given composite frequency than those that record from a mixed population or from only FF motoneurons, because of the difference in spike durations.

Figure 10 presents the calculated degree of spike overlap vs. frequency of firing of motoneurons across a set of simulations using several electrode and motor intent settings (Simulation run **4**). Figure 10A presents spike overlap as a function of motor intent for each of the electrodes. The plots demonstrate that percent overlap increases as a result of increased motor intent and the number of axons that contribute to a particular electrode. Note that the overlap in electrodes that record solely from S fibers reaches a plateau at motor intent = 0.5 (since this value was specified as the saturation point for that motor pool); the electrodes that record solely from FF fibers show overlap only for values of motor intent greater than 0.5 (since this value was specified as the threshold value point for that motor pool); and the electrodes that record from a combination of S and FF show a gradual increase in spike overlap throughout the range. Also note that the maximum values recorded for spike overlap was approximately the same for the three groups of electrodes (S, FF, and S & FF) due to the fact that the effect on spike overlap of lower firing rates of the S axons was offset by their longer spike durations. This effect is also demonstrated in Figure 10B, which demonstrates that overlap on electrodes that recorded from S axons increased more rapidly as a function of composite firing rate than those that recorded from FF axons; the rate of increase in overlap for the S & FF electrodes was at an intermediate level.

## Discussion

### A tool to facilitate the development of decoding algorithms

The purpose of this simulator is to facilitate the development of effective and reliable decoders for the control of prostheses by neural signals. Neural interfaces may improve the functionality of advanced prosthetic limbs and reduce the attentional demands required to operate them. Some of the key technical challenges in developing these neural interface technologies are to obtain a large number of independently controllable signals, to obtain them reliably and to interpret them appropriately. This work was directed at creating a tool to be used in the development of technology for interpreting, or decoding, the recorded neural signals.

In a neural controlled prosthesis, the role of the decoder is to estimate the intent of the user from the recorded neural signal. According to our general definition as well as our specific implementation, motor intent is a multi-dimensional signal that can take on graded values along each dimension. The recorded neural signals are a set of waveforms, each of which is a composite of spike trains from several motoneuron sources. In general, an increase in the intensity of motor intent along any dimension is likely to increase the level of activity on one or more electrodes. Therefore, one challenge for the decoding process is to identify changes in activity level in the recorded signals, which would indicate a change in the intensity of motor intent. A second challenge for the decoding process is to accomplish a mapping from a multi-dimensional space defined by electrode recordings to space defined by dimensions of motor intent.

Consider the first challenge—that of identifying changes in activity level on a given electrode. In electrodes that record composite signals, any overlap in the action potentials in neighboring axons will produce distortion in the morphology of a given spike. Some candidate decoding algorithms may be more sensitive than others to such distortions due to spike overlap. In evaluating a decoder on actual recordings from nerves, the amount of overlap is not known and cannot be experimentally controlled. The simulator described here will enable comprehensive assessment of candidate algorithms with respect to their ability to identify changes in motor intent and with respect to their sensitivity to distortions caused by spike overlap. The simulator can be used to generate data sets with a collection of motor intent signals and a variety of electrode configurations. These data sets can be created to present specific and well-characterized challenges for decoding, such as spike overlap, in order to assess the ability of the algorithm to address that specific issue.

Next consider the second challenge for the decoding process—to accomplish a mapping from a multi-dimensional electrode space to motor intent space. In the situation where there is cross-talk, i.e., when signals from two or more motor pools contribute substantially to the signal recorded by one electrode, the decoding algorithm must be able to identify both components of the signal. Once again, it is likely that some candidate algorithms would address this problem better than others and the simulator would facilitate a comprehensive comparison.

In both of these cases, these capabilities of the simulator are particularly important because it is not possible to perform such a set of experiments in humans or an animal model. Distortions due to spike overlap and cross-talk of several motor pools onto one electrode cannot be controlled experimentally nor can they be quantitatively identified when they occur.

### A model that captures the key features of recorded neural signals, yet can be efficiently simulated

Many previous reports have described the design and development of simulation systems for spinal motor pools (Capaday and Stein, 1987; Fuglevand et al., 1993; Bashor, 1998; Nussbaumer et al., 2002; Ivashko et al., 2003; Lowery and Erim, 2005; Subramanian et al., 2005; Stienen et al., 2007; Uchiyama and Windhorst, 2007; Cisi and Kohn, 2008) and models of recordings of extracellular potentials (Plonsey et al., 2007). To the best of our knowledge, these two types of models have not been integrated in a manner that would meet our stated needs. The models of spinal motor pools include several efforts directed at studying the neuromotor control system (Fuglevand et al., 1993; Ivashko et al., 2003; Rybak et al., 2006) and others directed at designing biomimetic control systems (Ijspeert, 2008). The models of neural recordings have focused primarily on understanding and optimizing the electrode-tissue interface (Perez-Orive and Durund, 2000). Although our model and simulation system draws upon many of the concepts implemented in previous studies, we did not directly implement these other models.

In designing the model and the simulator, our intent was to capture the key features of recorded signals that may differentiate the performance of various decoding algorithms in a system. For the overall structure and for the individual elements, there are clear tradeoffs between biological fidelity and operational efficiency. Models that have a high degree of biological fidelity can often incur high costs in terms of effort required to develop the software, effort required to configure the software for a simulation run, and computational complexity. In developing this system, we focused on the key features of biological fidelity while striving to achieve reasonable operational efficiency. The key features of the neural/electrode system that we believe are suitably captured include: gradation of motor intent, multidimensionality of motor intent, variability in firing rates of motor pools from different fiber types, recruitment properties of different fiber types, variability in spike morphology across motor axons and electrodes, jitter in spike train timing, superposition of spike trains from multiple motor axons onto one electrode, spike overlap, cross-talk from multiple motor pools onto one electrode, variability in the number and relative strengths of motor axons contributing to different electrodes, and noise superimposed on the relevant neural signals. These features are captured in a model that requires specification of parameters that affect the properties of the system in a straightforward manner. For example, in this system the user directly specifies the range of firing rates for a motor neuron of a particular type; in a model with a high degree of biological fidelity that included a model of the biophysical properties of the membrane and channel dynamics, the range of firing rates would emerge from the specification of a large number of interdependent model parameters and components. In this example, the model with higher biological fidelity would incur what we believe to be unnecessary costs in development, configuration, and implementation. We believe that the design of our simulator captures the key system features in a manner that is operationally efficient.

The transformation from motor intent to neural recordings certainly involves a large number of nonlinear, dynamic processes. The model we have implemented includes three nonlinear processes: the piecewise linear mapping from motoneuron state to mean firing rate, the spike event times based on motoneuron state, and the morphology of the spike template for a given neuron. All other processes involve linear transformations: the connectivity between motor intent and motoneuron activation, the convolution of spike events with spike templates, and the connectivity between motor axons and electrodes.

In neural recordings, the morphology of a recorded spike is influenced by the relative spacing (and orientation) of the electrode and the nodes of Ranvier as well as the electrical properties of the tissue. Alterations in the relative spacing, orientation or tissue properties could have a nonlinear effect on the spike morphology. As implemented, the system allows for linear scaling of the contribution of a motor unit to an electrode, but nonlinear effects that would modify spike morphology would have to be accommodated by a change in the spike template.

In a system that uses more than one electrode in a fascicle, it is possible that one neuron may produce signals that contribute substantively to the recordings on more than one electrode. In this scenario, the morphology of the spike templates from that neuron will be different on each electrode. As designed, our simulator allows for a scaled version of the same template on different electrodes, but it does not allow for one axon to produce different morphologies on different electrodes. This limitation, which may be particularly important if using the simulator to study recordings on densely packed intrafascicular electrode arrays, could be addressed by modifying the simulator to allow one point process to produce more than one simulated spike train, thus producing simultaneous spikes on different electrodes with different shapes.

### Specification of motor intent

In this simulator, we have implemented motor intent as a signal that has two essential components: an intended action and a level of effort. The intended action is the DOF to be controlled while intended effort is the intensity of that action. Motor intent could be used to represent an action that is formulated in joint torque space. That is, motor intent signals could be used to represent quantities such as elbow flexion moment or wrist abduction moment. We believe that this form of representation will directly facilitate translation to a system where an amputee controls a motorized prosthesis. There are many possible representations of motor intent (in task space, joint space, muscle space, or other body-referenced coordinate systems) and there is evidence to support the existence of such representations at various points in the neuromotor control system circuitry. We believe that the joint torque representation is suitable because it will directly transfer to a constrained experimental paradigm in which an amputee is asked to issue specific motor commands, and the motor commands are directly related to the required actions of the prosthesis. For example, if an amputee is asked to think about elbow flexion and wrist extension while seated quietly, motoneurons in the residual limb that used to innervate elbow flexors and wrist extensors are likely to fire. Subsequently, when attempting to perform a functional task with a neural-controlled, powered prosthesis those same motoneurons are likely to fire if the task requires elbow flexion and wrist extension. These recorded commands can then be directly mapped to motors on the prosthesis to execute the desired movement.

### On-going and future work

We are currently using the simulator to develop data sets that will be useful in comparative assessment of decoding algorithms for neural-controlled prostheses. Although our current effort is directed at systems that would utilize LIFE electrodes, we believe that the simulator can be readily configured to simulate recordings from a Utah array, tfLIFE, TIME, or other electrodes designed to record from peripheral nerves. The primary differences in configurations for the different electrode types would be alterations of the spike template morphologies, the number as well as the relative contributions of motoneurons that contribute to a recorded signal, and the noise characteristics.

Several modifications to the existing simulation system might enhance its utility as a tool to characterize the benefits of various decoder designs. For example, the system described here uses a linear function to map motor intent to motoneuron activation. While this may be sufficient to test most of the key features of the decoding system, it may fail to capture other influences on the transformation that may impact decoder performance. Future efforts will seek to identify such opportunities for improving the utility of the simulation system.

### Conflict of interest statement

The authors declare that the research was conducted in the absence of any commercial or financial relationships that could be construed as a potential conflict of interest.
